# Tumor Microenvironment as A “Game Changer” in Cancer Radiotherapy

**DOI:** 10.3390/ijms20133212

**Published:** 2019-06-29

**Authors:** Magdalena Jarosz-Biej, Ryszard Smolarczyk, Tomasz Cichoń, Natalia Kułach

**Affiliations:** Center for Translational Research and Molecular Biology of Cancer, Maria Skłodowska-Curie Memorial Cancer Center and Institute of Oncology, Gliwice Branch, Wybrzeże Armii Krajowej Street 15, 44-101 Gliwice, Poland

**Keywords:** tumor microenvironment, radiotherapy, “in situ” vaccination, immunosuppression, tumor vasculature, hypoxia, radioresistance

## Abstract

Radiotherapy (RT), besides cancer cells, also affects the tumor microenvironment (TME): tumor blood vessels and cells of the immune system. It damages endothelial cells and causes radiation-induced inflammation. Damaged vessels inhibit the infiltration of CD8+ T lymphocytes into tumors, and immunosuppressive pathways are activated. They lead to the accumulation of radioresistant suppressor cells, including tumor-associated macrophages (TAMs) with the M2 phenotype, myeloid-derived suppressor cells (MDSCs), and regulatory T cells (Tregs). The area of tumor hypoxia increases. Hypoxia reduces oxygen-dependent DNA damage and weakens the anti-cancer RT effect. It activates the formation of new blood vessels and leads to cancer relapse after irradiation. Irradiation may also activate the immune response through immunogenic cell death induction. This leads to the “in situ” vaccination effect. In this article, we review how changes in the TME affect radiation-induced anticancer efficacy. There is a very delicate balance between the activation of the immune system and the immunosuppression induced by RT. The effects of RT doses on immune system reactions and also on tumor vascularization remain unclear. A better understanding of these interactions will contribute to the optimization of RT treatment, which may prevent the recurrence of cancer.

## 1. Introduction

The tumor microenvironment (TME) is an “ecological niche” that stimulates the progression of cancer [1,2]. TME complexity is associated with tumor growth, metastasis, and response to therapy. Dynamic changes occurring in the TME cause tumor cell variants selection, which may promote genomic instability [1,3]. Cancer is an extremely heterogeneous disease. Cancer cells present in the tumor have different mutations at various sites within the primary tumor as well as in the metastases [4,5]. 

The TME consists of, among other things, tumor blood vessels and the cells of the immune system that inhibit the antitumor immune response [3,6,7,8,9]. Cells of the immune system include tumor-associated macrophages (TAMs) with the M2 phenotype, tumor-associated neutrophils (TANs) with the N2 phenotype, MDSCs (myeloid-derived suppressor cells), mast cells, and natural killer (NK) cells, producing a variety of factors (chemokines, cytokines, and enzymes) that directly or indirectly act as pro-angiogenic factors [8,10,11]. The emerging network of blood vessels is defective and functionally impaired [12,13,14,15,16,17]. It leads to the formation of hypoxia [10,16,18,19,20]. Hypoxia is an important metabolic element in TME that affects cell plasticity and tumor heterogeneity [21]. It is a regulator of cancer hallmarks [22,23]. Hypoxia induces an inflammatory reaction similar to the one present in damaged tissues [23,24]. In response to proinflammatory signals (chemokine (C-C motif) ligands 2 and 5 (CCL2 and CCL5), colony-stimulating factor-1 (CSF-1), vascular endothelial growth factor (VEGF), endothelial monocyte activating polypeptide II (EMAP II), and endothelins (ET-1 and ET-2)) the cells of the immune system are recruited to the TME and undergo specific “reprogramming”, e.g., monocytes differentiate into specific tumor-associated macrophages [25,26,27,28]. TAMs are involved in angiogenesis, immunosuppression, matrix remodeling, invasiveness, and metastasis [2,24,29,30,31,32]. The activation of an immunosuppressive environment promoting tumor growth also includes the inhibition of differentiation and maturation of dendritic cells (DCs), NK cell cytotoxicity, inactivation of proapoptotic pathways, inhibition of antigen presentation, disorders in receptor signaling of T cells, and activation of negative co-stimulatory signals like CTLA-4 (cytotoxic T-lymphocyte-associated protein 4)/CD80 (or CD86) and PD-1 (programmed death 1)/PDL-1 (programmed death ligand 1) [33]. 

**Figure 1:**
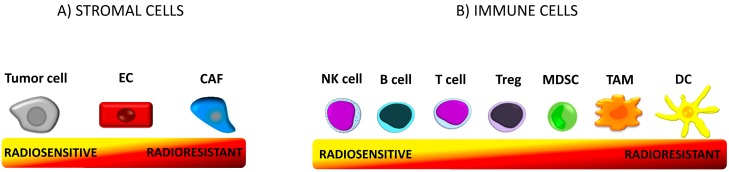
Tumor microenvironment (TME). TME is a functional and structural niche where tumor progression occurs. It consists of cellular and molecular (extracellular matrix, cytokines, chemokines, and other molecules) components. The microenvironment is composed of tumor stromal cells (cancer-associated fibroblast (CAFs), mesenchymal stromal cells (MSCs), endothelial cells (ECs), pericytes) and immune cells (T cells, B cells, natural killer (NK) cells, dendritic cells (DCs), tumor-associated macrophages (TAMs), tumor-associated neutrophils (TANs), myeloid-derived suppressor cells (MDSCs)) [6]. The cells differ in radiosensitivity. The term “radiosensitivity” means the relative susceptibility of cells to radiotherapy (RT)-induced irreversible damage such as chromosomal instability and cell death [37]. (**A**) Proliferating tumor cells are sensitive to irradiation (IR) [37]. Endothelial cells are resistant to doses up to 10Gy. CAFs are the most resistant stromal cells. (**B**) Within the cells of the immune system regulatory T cells (Tregs) are more radioresistant than any other population of T cells [38] and B cells [39]. NK cells and B lymphocytes are the most radiosensitive immune cells, while DCs are the most resistant [40].

The immuno-privileged TME is one of the main barriers to effective anticancer therapy [34]. Harrington and co-workers [35] relate the 5 Rs of radiobiology—repair, repopulation, radiosensitivity, redistribution, and reoxygenation—with the hallmarks of cancer (sustained proliferative signaling, evasion of growth suppressors, resistance to cell death, angiogenesis, unlimited replicative capacity, and invasive and metastatic phenotypes) [1]. In 2013, Good and Harrington added two new emerging hallmarks: evading immune destruction and altered energy metabolism [36]. Figure 1 shows schematic representations of the radiosensitivity of tumor microenvironment cells.

In the article, we try to indicate that the tumor microenvironment is a “game changer” in radiotherapy. Radioresistance remains the main reason for the failure of cancer patients’ treatments. However, a better understanding of the processes taking place in the TME under the influence of irradiation (IR) should be used to establish new, effective radiotherapy (RT) schemas and to associate RT with other available therapies such as immunotherapy, among others. 

## 2. Tumor Milieu as a Critical Immune Mediator of RT

### 2.1. RT as A Frontline Anticancer Therapy

Radiotherapy (RT) is the major method of treatment for cancer patients [33]. It is applied for approximately 60% of all newly diagnosed patients as a frontline therapy [41]. The effectiveness of radiotherapy is interpreted as a consequence of better local tumor control and reduction of the spread of the disease [42,43]. In comparison to chemotherapy, RT shows better effects in local tumor control, with fewer side effects [44]. RT is used in the treatment of many types of cancers and also as a complementary therapy to prevent recurrence (irradiation of the surgical cavity after tumor excision) [45]. It is also routinely used in palliative treatment to reduce symptoms and increase the quality of life of patients with advanced cancers [41,45]. Radiotherapy is the most effective cytotoxic therapy available for the treatment of patients with solid tumors [46]. It increases disease-free survival in treated patients. The overall 5-year survival rate in the USA is over 65% [47]. 

Radiation therapy as one of local ablative physical therapies [34] uses high energy radiation for local cancer treatment [48]. It induces double-strand DNA damage in cancer cells [49,50], single strand breaks [51], misrepair, and chromosome aberrations [34]. All of these events are direct actions of RT [44]. The cells are killed mainly by mitotic catastrophe, but also by apoptosis, necrosis, autophagy, or replicative senescence [49,52,53,54,55]. 

### 2.2. Immunogenic Cell Death in Irradiated TME

In addition to the direct destruction of cancer cells, irradiation also activates the immune response [52,56]. This phenomenon is called “in situ” vaccination [41,56,57,58,59]. Stimulation of the anti-cancer immune response is a paradigm shift in oncology [5,60]. RT participates in the modulation of many immunological processes: release and presentation of antigens, priming and activation of T lymphocytes, recruitment and accumulation of T cells in the tumor, recognition and killing of tumor cells by T lymphocytes [61]. Factors released from dead cells may be the source of radiation-associated antigenic proteins (RAAPs) [44]. Cross-priming of T cells induced by RT depends on the activation of immunogenic cell death molecular signals and requires the production of interferon (IFN) I by tumor-infiltrating cells [62]. Immunogenic cell death induces (locally and/or systemically) a release of tumor-associated antigens (TAAs) [63]. Dying cells actively or passively release cell death-associated molecular patterns (CDAMP), which are captured by appropriate pattern recognition receptors (PRR) on immune cells infiltrating tumors. This initiates a “dialogue” between dying cancer cells and the immune system and thus the stimulation of immune surveillance [34,64].

### 2.3. Innate and Adaptive Immune Response Activation

Irradiation exerts an immunostimulating activity by increasing NK cell cytotoxicity and tumor infiltration by CD8+ cytotoxic T lymphocytes, the accumulation of tumor-associated M1 macrophages (inhibiting tumor growth), reducing the level of infiltrating regulatory T cell (Treg) lymphocytes [65], enhancing the expression of Fas and IFN-γ, and the inhibition of the PD-1/PDL-1 pathway [66]. Transformation of the TME is an indirect effect of RT [44]. In addition, irradiation increases the amount of major histocompatibility complex (MHC) molecules on the surface of the cells [34], causes the translocation of calreticulin (CRT), the release of high mobility group box 1 (HMGB1) protein [67,68], and the secretion of adenosine triphosphate (ATP) and heat-shock proteins (HSPs) [34]. These signals are necessary for the activation of dendritic cells that initiate innate and adaptive immune responses [48]. DCs are not potent antigen presenting cells (APCs), and factors secreted after RT increase their ability to present antigens [47]. ATP is involved in the recruitment of monocytes to the tumor (via the P2Y2 receptor) and participates in the production of interleukin (IL)-1β (through the P2RX7 receptor present on DCs and the activation of inflammasome NLRP3) necessary for the activation of T cells [34]. Hsp70, translocated from the cytoplasm to the extracellular matrix, activates monocytes, macrophages, and DCs by binding to CD14, CD40, CD91, Lox1, and Toll-like receptors (TLR2 and TLR4) [37]. CRT, by binding to CD91 receptors present on DCs or macrophages, stimulates the phagocytosis of TAA and its presentation on MHC-1 [69,70]. Furthermore, IR upregulates the NK pathway by the activation of NKG2D ligands [33,70], increases NK cell cytotoxicity, tumor infiltration, and the production of many cytokines [71].

### 2.4. IR-Induced T Lymphocytes Activation

Activated DCs migrate to the lymph nodes, where they activate T lymphocytes [61,67]. After irradiation costimulatory molecules (CD80), adhesion molecules (like intercellular adhesion molecule 1 (ICAM-1)), or stress ligand (NKG2DL) levels are increased on the surface of tumor cells [42,48,72]. Additionally, increased expression of MHC-I, TAA, and the Fas/Fas ligand pathway make tumor cells more sensitive to cytotoxic T lymphocyte attack [41,49,71,73]. In addition, HMGB1 released from dying cells stimulates the TLR4/MyD88/TRIIF pathway, which activates T cells [33]. 

RT activates pro-inflammatory factors including interferons and chemokines that attract activated T cells into tumors [41]. IR induces the C-X-C motif ligand (CXCL)9 and CXCL16 chemokines that recruit both effector CD8+ T cells and helper CD4+ T cells [33]. The increased level of retinoic acid early transcript 1 (RAET1) protein on the surface of tumor cells affects the formation of productive immunological synapses [74]. IR also induces the production of IFN (type I) by activating a stimulator of interferon genes (STING) pathway in tumor-infiltrating DCs, as well as the increased secretion of CXCL10, a C-X-C chemokine receptor (CXCR3^+^) that recruits IFN-γ secreting CD8+ T cells [34,75]. STING protein is activated by cyclic GMP-AMP (cGAMP) produced by cGAMP synthase (cGAS), which detects dsDNA fragments in irradiated cancer cells [34,45,70,76]. Binding of cGAMP by STING activates a number of transcription factors like NF-κB, IRF3, IRF7 STAT3, and STAT6, which stimulate the immune system to respond against pathogens and cancer cells [77,78]. Type I IFN stimulates DCs to present tumor-associated antigens to T lymphocytes, thereby activating the specific T-cell response both within the irradiated site and in the lymph nodes. Activated T cells and NK cells secrete type II IFN, i.e., IFN-γ, which triggers the expression of MHC-I on the surface of tumor cells [52,70]. 

### 2.5. IR-Induced Tumor Infiltration of Immune Cells 

RT affects leukocyte infiltration into the tumor by three different mechanisms: a change in vascular structure, increased expression of adhesion molecules, and chemokine secretion [79]. The increased production of cellular adhesion molecules contributes to the influx of antitumor T cells [40]. In addition, IR-induced inflammatory cytokines—IL-1β, tumor necrosis factor (TNF)-α, and type I and II IFNs—affect the upregulation of vascular cell adhesion molecule 1 (VCAM-1) on tumor endothelium [42,80,81]. Increased expression of adhesion molecules—intercellular adhesion molecule 1 (ICAM1) and VCAM1—in tumor vessels enables tumor infiltration by T lymphocytes [41,47]. ICAM-1 also mediates the migration of neutrophils into the tumor [80]. IR induces the rapid and transient infiltration of neutrophils that eliminate tumor cells by releasing reactive oxygen species (ROS) [34].

### 2.6. Systemic Reaction of Irradiated TME

The TME is a critical mediator in response to IR, both locally and systemically [82], therefore RT may have also an inhibitory effect on cancer cells outside the irradiation site [83,84]. This includes the bystander effect (when the signals from irradiated cells affect neighboring non-irradiated tissue responses) [51,61,85,86,87]. An example is the direct IR effect on the activation of macrophages, which then produce bystander signals and play an important role in the development of radiation injury [88]. An abscopal effect, defined as “an action at a distance from the irradiated volume but within the same organism” is also observed [70,85,89,90,91,92,93]. The abscopal effect is an immune system response [69,94]. It can be mediated by DCs and macrophages, activated by inflammatory agents (cytokines, DAMPs, reactive oxygen/nitrogen species (ROS/RNS)) originating from irradiated TME. These cells migrate to cancer lymph nodes and distant non-irradiated sites [95]. Additionally, the cGAS /STING pathway in irradiated cancer cells stimulates the production of IFN I, which is critical in the abscopal response. It takes part in the activation of BATF3-DCs that migrate to lymph nodes (LNs), prime CD8 T cells, and activate the cytotoxic response [45]. After the activation in the lymph nodes, NK cells, CTL (cytotoxic T lymphocytes), and Th cells migrate to distant tumor sites. There, through a pro-inflammatory response, they lead to tumor suppression in non-irradiated tumors [95]. RT may also induce a local inflammatory reaction, leading to the activation of T-cell responses against tumor antigens. After CTL activation, they migrate not only to the irradiated tumor, but also to distant metastasis sites and may be responsible for the abscopal effect [45]. Within the irradiated TME, activated T cells also secrete a number of cytokines that participate in eliciting tumor immunosurveillance and its growth inhibition, which in consequence triggers an abscopal effect. For example, TNF produced by IR-activated T cells leads to the direct elimination of MDSCs both locally and systemically [76,96].

## 3. Immunosuppressive TME as a Side Effect of RT 

### 3.1. Immunostimulation Processes Activate Radioresistance 

Paradoxically, some of the processes taking place in the TME that are involved in the stimulation of the immune system may also participate in the activation of immunosuppression and induction of radioresistance: 1) Activation of STING after irradiation may activate the recruited MDSC through the CCR2 (C-C chemokine receptor type 2) pathway [68]. 2) Activation of noncanonical NFκB (nuclear factor kappa-light-chain-enhancer of activated B cells) pathway through the cGAS-STING DNA sensing pathway may also inhibit IL-1β expression in dendritic cells [97]. 3) The secretion of type I and II IFNs may elicit the upregulation of PD-L1 on cancer cells and the immune system cells. Upregulation of PD-L1 expression on tumor cells blocks the antitumor function of activated T and NK cells [33]. 4) T cells, which can also overexpress PDL-1 after radiation, contribute to the prevention of tumor cell recognition [47]. In addition, RT may induce a downregulation of co-stimulatory CD80 and CD86 molecules present on immature DC cells, hindering the activation of T cells [70]. Furthermore, NK cells may also be inactivated by the RT-driven exposure of MHC I molecules on the surface of tumor cells [98]. Increased apoptosis of RT-induced tumor cells can lead to tolerogenic DC appearance, which in turn induces the suppressive Treg lymphocyte population [39].

### 3.2. Immunosuppressive Pathways in Irradiated TME

After irradiation, immunosuppressive pathways are activated in the tumor microenvironment [49,65]. Inflammatory switchover within the irradiated TME can trigger processes responsible for the formation of metastases. Unfortunately, despite the increasing effectiveness of RT (related mainly to the increased precision of irradiation), second cancers as “late effects” of RT appear. Radiation-induced inflammation is caused by the activity of MAPK family members—JNK, P38, ERK1/—and DNA-repair pathways—ERCC1, XRCC1, XPP, ATM. The translocation of Ap-1, NF-κB, and IRF-3 into the cell nucleus induces the transcription of proinflammatory cytokines [47]. Pro-inflammatory factors affect tumor progression by stimulating tumor cell proliferation and influencing genomic instability by enzymes responsible for somatic hypermutation [34]. In these processes, immunosuppressive cells are involved, namely Tregs, tolerogenic and immunosuppressive DCs, TAMs, TANs, MDSCs, and a number of molecules such as transforming growth factor-β (TGF-β), adenosine, VEGF, CSF-1, and CCL2 [56,99]. Activated TGF-β inhibits the radiosensitivity of tumor cells and may hinder the generation of “tumor vaccine” by RT [62]. This factor enhances immunosuppression by reducing CD8+ T cell cytotoxicity, inhibiting CD4+ T cell differentiation, promoting Treg transformation, and inhibiting NK cell proliferation [68]. It also induces the transformation of neutrophils towards the pro-tumor N2 phenotype and also the activation of Pl3K-Akt (phosphoinositide 3-kinase—v-akt murine thymoma viral oncogene homolog 1), RHOa (ras homolog gene family, member A), MAPK (mitogen-activated protein kinases), and SMAD (contraction of Sma and Mad (Mothers against decapentaplegic) pathways [33]. In addition, the increased expression of CSF-1, the cytokine responsible for macrophage polarization towards the M2 phenotype, as well as the presence of Tregs and MDSCs, maintains immunosuppression in TME [100]. Adenosine, lactate, potassium, and acidosis block the antitumor immune response. Suppression of T cells activity is also caused by the increased levels of immunosuppressive agents in irradiated TME, namely NOS (nitric oxide synthase), RNI (reactive nitrogen intermediates), ROS, IL-4, IL-10, MMPs (matrix metalloproteinases), LOX (lipoxygenase), Arg (arginase) 1, and collagenase [33].

### 3.3. IR-Induced Immunosuppressive Immune Cells

Within a few hours after irradiation, granulocyte–macrophage colony-stimulating factor (GMC-SF) stimulation occurs, which promotes the migration of MDSCs to the circulatory system and to inflammatory tissue [49]. RT also induces the C5a molecule, which is a classical inducer of MDSCs. MDSCs show radioprotective activity. They produce high levels of Arg1, which promotes tumor progression through the degradation of arginine, an essential amino acid in the activation and function of T cells [99]. Arg1 also reduces the expression of the zeta chain of the CD3 complex and thus weakens T cell activity [33]. MDSCs may also limit the availability of cysteine (an amino acid necessary for T cell proliferation) and produce ROS that destroy T cell receptors [101]. They can also trigger the PDL-1 pathway or IL-10 secretion [56]. 

MDSCs can differentiate into mature granulocytes and macrophages [49]. Tumor-associated macrophages may exhibit pro- (M2 phenotype) or anti-tumor properties (M1 phenotype) [31,49,102]. M2 macrophages induce Treg lymphocytes and T-cell responses without antitumor activity. M1 macrophages stimulate naїve T cells to elicit a Th1/cytotoxic response [27,28,31,46]. While M2-like cells participate in the formation of abnormal dysfunctional blood vessels, M1-like cells tend to normalize tumor blood vasculature [19,103,104,105]. Apoptotic cells, appearing after RT, activate macrophages with the M2 phenotype to secrete a series of anti-inflammatory cytokines such as TGF-β and IL-10, among others [33]. Hypoxia causes HIF-1α-dependent up-regulation of PD-L1 on TAMS, which leads to the anti-tumor immune response suppression [106].

In addition, the presence of Tregs may affect the effectiveness of radiotherapy [39]. Irradiation increases the level of Tregs, thus limiting the positive, immunomodulatory effects of hypofractionated RT. A release of adenosine by tumor cells, as well as the increased regulation of TGF-β, is involved in Treg accumulation after irradiation [41]. The presence of chemokines CXCR3, CCL10, CXCR4, and CCL7 is also involved in Treg recruitment in the TME [39]. Tregs are more radioresistant than other T and B cell subpopulations [39,107]. They show increased expression of Akt, making them more resistant to IR-induced apoptosis. Tregs constitute a highly suppressive cell population that expresses ectonucleotidases CD39 and CD73 capable of hydrolyzing ATP. Tregs survive IR and inhibit effector cell proliferation [39]. Regulatory T-lymphocytes block the activation of T-lymphocytes by the high expression of CTLA4 [56]. Table 1 lists the factors activated in the TME in response to RT, which contributes to tumor radioresistance (acc. [47,65]).

### 3.4. CAFs in Irradiated TME

Radiation injury strengthens the proinflammatory response within the TME and recruits stromal CAFs—fibroblasts that promote tumor growth [66]. In irradiated TME myofibroblasts also undergo phenotypic transformation to CAFs. CAFs, activated by irradiation, secrete a number of cytokines, growth factors (hepatocyte growth factor (HGF), TGF-β, platelet-derived growth factor (PDGF)), chemokines (CXCL12), extracellular matrix (ECM) proteins (tenascin C (TNC), collagen I), and modulators of the extracellular matrix (matrix metalloproteinases (MMPs)) [47]. CAFs can induce autophagy and recovery of irradiated cancer cells by insulin-like growth factor 1-mediated mechanisms. They also produce collagen, fibronectin, and integrins. Expression of integrins is strongly associated with radioprotection and increases the proliferation of tumor cells [82]. Through secreted SDF-1 (stromal cell-derived factor 1), they promote bone-marrow-derived dendritic cell (BM-DC) recruitment, which is involved in the process of vasculogenesis [3]. CAFs also recruit endothelial progenitor cells for the formation of new blood vessels and participate in the recurrence of cancer [66]. 

## 4. Tumor Vasculature and RT

Immunosuppressive properties of TME promote the destruction of blood vessels, which in turn limits the infiltration of cytotoxic T lymphocytes into the tumor and increases the hypoxia [98]. Reconstruction of vessels is a hallmark of IR damage [55]. Understanding the effect of radiotherapy on the functionality of tumor vasculature is important to maximize the effectiveness of radiotherapy [56]. 

### 4.1. IR-Induced EC Dysfunctions

The emerging vessels are often devoid of a basement membrane and pericytes, making them more permeable, leaky, and sensitive to irradiation than the vessels surrounding healthy tissues. The fast rate of endothelial cell proliferation makes them sensitive to RT. Irradiation induces endothelial cell dysfunction characterized by increased permeability, detachment from the basement membrane, and apoptosis. This contributes to the development of radiation-induced inflammation and fibrosis [56]. IR-induced endothelial cell apoptosis can lead to vascular destruction, indirectly leading to tumor cell death [3]. IR may also induce endothelial cell (EC) senescence [108]. Changes occurring in aging EC cells lead to endothelial dysfunction, which results in the suppression of angiogenesis, induction of oxidative stress, and inflammation [109]. Both senescent and apoptotic cells secrete cytokines that may contribute to long-term vascular dysfunction. The death of ECs under the influence of IR also induces anti-tumor signals—TNF cytokine, which activates macrophages; CXCL6 chemokine, which recruits immune cells, and signals that activate Toll-like receptors on DCs [110]. The vascular endothelium acts as a barrier regulating the rolling of immune cells on the vascular surface and may be the main control point for IR-induced immune responses [111]. 

### 4.2. Pro-Survival Processes in the TME after EC Irradiation 

After irradiation, in tumors, a number of pro-survival cytokines are secreted. The cytokines inhibit apoptosis of ECs, prevent vascular damage, and weaken the anti-cancer RT effect [112]. EC irradiation may trigger pro-survival processes including the overexpression of αγβ3 integrin, Akt phosphorylation, upregulation of angiogenesis mediated by vascular endothelial growth factor receptor 2 (VEGFR2) and basic fibroblast growth factor (bFGF) [47]. The secretion of VEGF and bFGF by tumor cells promotes the survival of endothelial cells, and the maintenance of vascular functionality increases the survival of cancer cells [113]. The proangiogenic effect of RT is induced by the expression of factors Bv8, S100A8, TGF-β, and VEGF [33]. Monocytes recruited into tumors can repair IR-damaged vasculature by expression of MMP-9, S100A8, or by the release of VEGF [101]. The increased presence of adhesion-associated surface proteins and the IR-induced adhesion of tumor cells to endothelial cells may contribute to the formation of metastases [114].

### 4.3. Vascular Remodeling in Irradiated TME

Vessels’ sensitivity to IR correlates with their morphology—capillaries and small, immature vessels are extremely sensitive, while larger, mature ones are more resistant to RT [13,20,110]. Reduced vascular density increases the distance between functional vessels, resulting in less efficient tissue perfusion. Blood vessels become thicker and prone to atherosclerosis. Subsequent morphological changes include thrombosis, fibrosis and medial necrosis [56], telangiectasia and capillary rupture, and indicate local vascular dysfunction [13]. IR can inhibit ongoing angiogenesis (fast-proliferating endothelial cells) without affecting mature quiescent vessels [13].

### 4.4. IR-Induced Vasculogenesis

Cancer cells release factors recruiting circulating endothelial precursor cells (EPCs) or BM-derived hematopoietic cells for vasculogenesis. The switch from angiogenesis to vasculogenesis is supported by the activation of the stromal-cell-derived factor-1 (SDF-1) receptor (CXCR4) and stabilization of HIF1 [47]. Vasculogenesis is the main mechanism for repairing the vessel network and re-growth of the tumor after RT [115,116]. MMP-9-expressing BM-derived myelomonocytic cells, not EPCs, are involved in these process of tumor revascularization [13,117,118].

### 4.5. Hypoxia in Irradiated TME

The destruction of vascularization by irradiation increases hypoxia in tumors. Hypoxia reduces oxygen-dependent DNA damage [56,119]. Hypoxia is a key regulatory factor for tumor growth that plays a critical role in radioresistance [56]. It correlates with tumor recurrence and poor prognosis after RT [47]. Hypoxia increases radioresistance more than three times. Hypoxia supports cancer stem cells at dormancy, preserving their potential for proliferation and differentiation, thus protecting them from radiotherapy [119]. Cancer cells are much more resistant to IR in hypoxic conditions [120]. Signaling pathways involved in the processes of adaptation to hypoxia change the phenotypes of cells and enhance their resistance to IR [121]. Hypoxia also inhibits the expression of many markers of differentiation and maturation, namely CD1α, CD40, CD80, CD83, CD86 and MHC II, involved in the development of the anticancer immune response. Damaged vessels block tumor infiltration by CD8+ T lymphocytes [65], and hypoxia inhibits their proliferation and induces the production of immunosuppressive cytokine IL-10 [101].

Hypoxia induces HIF-1α-mediated cell survival [56]. Activation of HIF-1α increases the expression of enzymes that participate in glycolysis and influence the accumulation of lactate and pyruvate, glutathione, and NADPH (nicotinamide adenine dinucleotide phosphate). Lactate increases tumor resistance [97]. HIF-1 also increases the activity of the serine synthesis pathway and pentose phosphate pathways. This, in turn, increases the production of antioxidants neutralizing (buffering) ROS induced by IR and causes radioresistance [119]. The resulting hypoxia recruits immune cells [47], including TAMs with the M2 phenotype [25,46,67,82]. Hypoxia promotes tumor angiogenesis and tolerance by inducing the expression of the chemokine CCL28, which recruits Tregs to the tumor [21,122]. In addition, the raised production of prostaglandin E2 (PGE2) and TGF-β increases the immunosuppressive populations of MDSC and Treg cells [67].

## 5. Different Responses of the TME after Various Doses of RT

### 5.1. Radioresistance of Immune Cells

An appropriate treatment regimen may play a key role in creating a specific immune response [49]. Depending on the dose, the method of administration, and the RT regimen, a different immune response is obtained in preclinical studies and clinical observations [123]. Ionizing radiation, depending on a dose, recruits and activates various types of immune cells [124]. The sensitivity of T-lymphocytes to radiation depends on the state of their activation—resting (non-activated) lymphocytes are much more affected by IR than their activated forms [125]. Tregs are more resistant to IR than other human T cells [43]. B-lymphocytes, on the other hand, show high radiosensitivity and have a reduced ability to present antigens and produce antibodies after IR [95]. Macrophages are more resistant to IR than monocytes [126]. However, human DCs under very high IR doses (up to 30 Gy) show only small phenotypic changes [53]. 

### 5.2. Immune Response in TME vs. Low Dose of RT

It seems that a low dose is unable to induce an effective antitumor response. A high dose, on the other hand, is more effective in triggering both innate and adaptive antitumor responses in murine tumor models (colon, lung, and melanoma) [127]. The results presented by various groups are very divergent. Low doses of irradiation induce biological reactions, such as inflammatory reactions, innate immune activation, and DNA repair (adaptive response) [37]. Administered on the whole body of melanoma-bearing mice, it reduces the level of Tregs and increases the effector-memory T cell frequencies [128]. Low IR doses may also induce the expression of ICAM-1 and E-selectin on endothelial cells to promote immune cell extravasation into the TME [100]. A single dose of about 2Gy may recruit tumor-specific CD8+ and CD4+ T lymphocytes into the tumor [83,124]. It may also induce vascular normalization by reprogramming the macrophage phenotype from M2 to M1 in treated mice [129]. However, the optimal stimulation of adaptive immunity may require three times larger fractions than conventional 2Gy. Preclinical mouse models indicate that these higher doses may be needed to release sufficient amounts of neoantigens, DAMPs and immunostimulatory molecules [46]. It was also shown that only fractionated, but not single, doses of RT can induce the immune-mediated abscopal effect [83]. 

Low doses may also activate immunosuppression and angiogenesis. In mice, after a low dose of radiation, M2 macrophages suppress the antitumor response and promote metastasis through the production of arginase and cytokines TGF-β and IL-10. Low doses can also induce the production of protumor cytokines such as IL-17A via IL-6 and TGF-β. Mast cells, after a low dose of radiation, release VEGF, which participates in angiogenesis and vasculogenesis [47]. Conventional fractional radiotherapy increases the amount of MDSCs, while the hypofractionated ablation dose reduces their levels in treated mice [99]. The fractional dose of RT (2 Gy/day), but not a single high dose of IR, increases the expression of immunosuppressive PDL-1 molecules on tumor cells [60]. Clinical observations showed that a dose < 4 Gy leads to cell death but a high dose induces a danger signal release, which activates the adaptive immune response, and at the same time, immunosuppressive signals like TGF-β [61]. 

### 5.3. Immune Response in TME vs. High Doses of RT

Preclinical studies showed that high doses per fraction > 8–10 Gy are more effective in increasing the antitumor response [70]. A low dose triggers apoptosis of cancer cells, while a high dose promotes necrosis [127]. A higher, single dose of ionizing radiation, which is used e.g., in hypofractionated radiotherapy, leads to the induction of immunogenic death of tumor cells [66]. In mice, a high dose of RT (12–18Gy) activates the DNA exonuclease Trex1, degrading cytosolic DNA, which is the main immunogenic trigger [60]. An immune response with type I IFN is also generated, which is involved in the activation of antigen-presenting cells including DCs [124,130]. One high dose affects the maturation of APCs and increases the infiltration of immune cells into the tumor [97]. A dose > 7Gy increases IFN production, and hypo-fractionated stereotactic body radiation therapy (SBRT; single fraction 20–24 Gy) causes the massive release of antigens, DAMP (death-associated molecular patterns) ligands, and TLR (Toll-like receptors) stimulation on APC cells [61]. Moreover, a single dose of 15 Gy induces an antitumor response in melanoma-bearing mice by increasing the level of APCs and IFN-γ production in the lymph nodes. Ablation doses of 15–20 Gy cause DC maturation, migration, and increase the level of tumor-reactive T cells [101]. They also increase the stimulation of T lymphocytes in murine lymphoid tissues [53]. 

The therapeutic effect of high doses of radiotherapy may also be due to the reduction of CAF levels in the tumor [66]. However, CAFs are radioresistant and capable of surviving at doses up to 50Gy in cell culture. A dose > 10 Gy induces irreversible changes in DNA and stress-induced cellular senescence. CAFs are metabolically active, secrete SASP (senescence-associated secretory phenotype) factors (growth factors, proteases, inflammatory mediators, or extracellular matrix proteins) and regulate tumor growth [127,131]. In addition, high doses of IR (>8 Gy) may promote the anti-inflammatory activation of macrophages [88], and a dose of 20 Gy activates the M2 phenotype of TAM with tolerogenic properties by induction of immune inhibitory molecules COX-2/PGE2 and NO [127,132]. 

### 5.4. EC Response vs. Doses of RT

The effect of IR on ECs is also dose-dependent [13,109]. IR induces endothelial cell dysfunction characterized by increased permeability, detachment from the basal basement membrane, and apoptosis [40]. Endothelial cells survive the conventional dose of 2 Gy [66]. In contrast, high doses induce the apoptosis of endothelial cells through the direct destruction of DNA and ceramide signaling [66,109,110]. A high single dose (8–16 Gy) increases the expression of acid sphingomyelinase (ASMase). This contributes to post-irradiation inflammation and fibrosis. Within the vessels, IR generates a prothrombotic state characterized by platelet aggregation, microthrombosis formation, and increased adhesion of inflammatory cells to endothelial cells with subsequent diapedesis to the perivascular space [40]. Exposure of endothelial cells to radiation doses of >0.5 Gy or <10 Gy primarily causes the senescence of ECs [109]. 

### 5.5. Vascular Response in TME vs. Low Dose of RT

Changes in vascularization after irradiation depend on the total dose, fractionation, type, location, and stage of the tumor progression [56]. Low doses of RT can normalize vessels, which facilitates the infiltration of T-lymphocytes into the tumor and the anti-tumor response [33,97]. The low-dose regimen may also stimulate the process of angiogenesis and neovascularization [66], while high doses might hamper these processes [13]. Because proangiogenic IR effects in low doses seem to be rapid and transient, daily administration of 2 Gy may repeatedly stimulate the angiogenesis process [13]. During fractionated radiotherapy, “tumor reoxygenation” may also occur when hypoxic cancer cells obtain better access to oxygen. Better distribution of oxygen in the vessels after previous fractions contribute to increased tumor cell death [127,133]. 

### 5.6. Vascular Response in the TME vs. High Dose of RT

**Figure 2:**
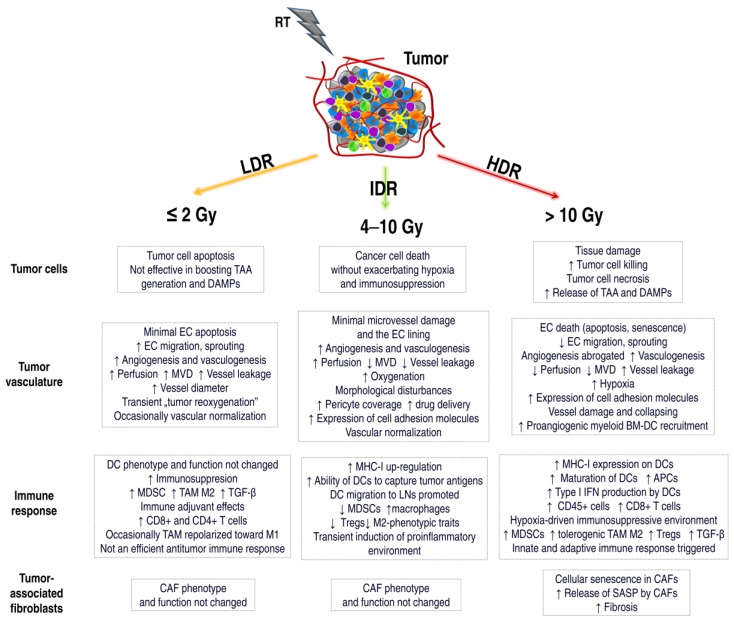
The effect of various doses of radiotherapy (RT) on the components of the tumor microenvironment. Radiation doses affect the cancer cells and the surrounding tumor microenvironment differently, including tumor vascularization, immune system cells, and CAFs. Low doses (Low-dose radiation, LDR) induce mainly apoptosis in cancer cells, with tolerogenic or immunogenic cell death. APCs are not activated, immunosuppressive macrophages TAM M2 and MDSCs are recruited. In some cases, TAMs may be polarized towards M1, and CD8+ and CD4+ T lymphocyte infiltration may be increased. However, the activated anticancer response is insufficient. ECs survive low IR doses, and angiogenesis/vasculogenesis is stimulated. During fractionated radiotherapy, “tumor reoxygenation” may occur, which leads to an increase in the effectiveness of RT. Intermediate-dose radiation (IDR) induces tumor cell death without increasing hypoxia or immunosuppression. MHC-I up-regulation, antigen presentation by DCs, reduced levels of MDSCs or Tregs, and transient induction of environmental proinflammatories occur. The vessels may be normalized, and perfusion, oxygenation, and the number of pericytes may be increased. IDR can also induce the process of angiogenesis or vasculogenesis. High-dose radiation (HDR) induces necrosis of tumor cells, and immunogenic cell death associated with the release of TAAs and DAMPs. An effective antitumor immune response is activated. ECs undergo apoptosis or senescence. Tumor vascularization is destroyed. Increased areas of hypoxia lead to an immunosuppressive environment. New vessels are formed in the process of vasculogenesis. CAFs also undergo a senescence process. They secrete a number of SASP factors involved in fibrosis and TME modulation. The effect of doses on tumor vascularization or immune system reactions is not entirely clear. There are conflicting literature reports. This is related to the fact that there is a delicate balance between the activation and inhibition of the immune system induced by RT. Further research into TME mechanisms triggered by various RT doses is necessary. TAA, tumor-associated antigens; DAMPs, death-associated molecular patterns; MVD, microvessel density; TGF-β, transforming growth factor-β; MHC-I, major histocompatibility complex I; LNs, lymph nodes; BM-DC, bone-marrow-derived dendritic cell; SASP, senescence-associated secretory phenotype.

Destruction of vascularity is mainly observed in doses greater than 5 Gy to 10 Gy. Exposure to one low dose of radiation initially causes an increase in blood flow in the neoplastic vessels and a rapid return to the level before irradiation [66]. While a single high dose (>10 Gy) destroys the vessels and drastically reduces blood flow. Thus, it changes the level of tumor oxygenation, which indirectly induces cell death, and as a result, reduces tumor volume [66,97,127]. Damaged vessels increase the areas of hypoxia in the irradiated tumor [127]. Lack of reoxygenation during hypofractionation of radiotherapy causes hypoxic tumors to be more resistant to IR [97]. A dose ≤ 5 Gy stimulates angiogenesis and/or vasculogenesis [127]. Irradiation with a 6 Gy dose increases the expression and activity of endothelial nitric oxide synthase (eNOS). It activates the nitric oxide (NO) pathway in ECs and induces tumor angiogenesis. Activation of the SDF-1/CXCR4 pathway is also increased. Recruitment of MDSCs and macrophages is involved mainly in vasculogenesis and promotes tumor re-growth [112]. One high dose of local IR activates two routes of BM-DC inflow. The first is fast (3–5 days after RT), and the second is a delayed response associated with increasing hypoxia, occurring after about 2 weeks. BM-DC recruitment is the main mechanism for rebuilding vessels after RT and is proportional to the IR dose [116]. Unfortunately, even after using the ablation dose, the blood flow can be reconstituted several weeks after the end of the RT regimen. The vascular effect may contribute 19–33% to the overall effect from single high-dose (20Gy) radiosurgery [127]. Figure 2 highlights the IR-induced TME reaction after different doses of RT [13,37,66,116,127,133,134].

## 6. Conclusions

Despite the many studies conducted, there is no clearly determined optimal dose and RT regimen in cancer therapy [49]. There is a very delicate balance between the activation of the immune system and the immunosuppression induced by RT, which is dependent on a specific radiation dose and fractionation scheme [42,60]. There are no unambiguous data showing which radiation doses affect the activation of the anticancer immune response and which lead to the development of immunosuppression. Literature reports show contradictory information—both LDR and HDR can activate as well as inhibit the antitumor immune response. Furthermore, the effect of doses on vascularization is not entirely clear. Further studies of TME mechanisms triggered by various RT schemes are needed to design effective therapeutic regimes [135]. In addition, our current understanding of RT as a therapy in systemic disease is limited [82]. In 2019, about 100 clinical trials are under way using anti-PD1/L1 immunoradiotherapy, although little is still known about how fractionation regimens, timing, and dosage of RT affects the anticancer immune response [33]. More knowledge on the effects of dose and fractionation of irradiation on TME cells may help in the optimization of treatment using RT [66].

In summary, technological progress allows for the irradiation of tumors with increasing precision, saving surrounding healthy tissue. However, despite the increasing precision and effectiveness of RT, recurrences of the neoplastic disease occur. The TME plays a key role in this process. Low doses slightly activate the antitumor response, without significant impact on the cancer blood vessels, which does not damage the entire tumor. High doses activate the antitumor immune response but also cause destruction, with huge regions of hypoxia that trigger renewal processes that lead to a regrowth of the tumor. In both cases, cancer may recur. Therefore, further studies investigating the effect of dose and RT schema on the TME are necessary. The acquired knowledge should be used to develop a treatment regimen that will destroy cancer cells and use the surrounding environment to effectively fight cancer. The key is to understand that TME is a “game changer” in the RT fight against cancer. Ignoring this information leads to therapy failure. Following these may lead to designing an effective combination therapy—radiotherapy with drugs that will prevent the unwanted, negative changes occurring in the tumor microenvironment.

## Figures and Tables

**Table 1:**
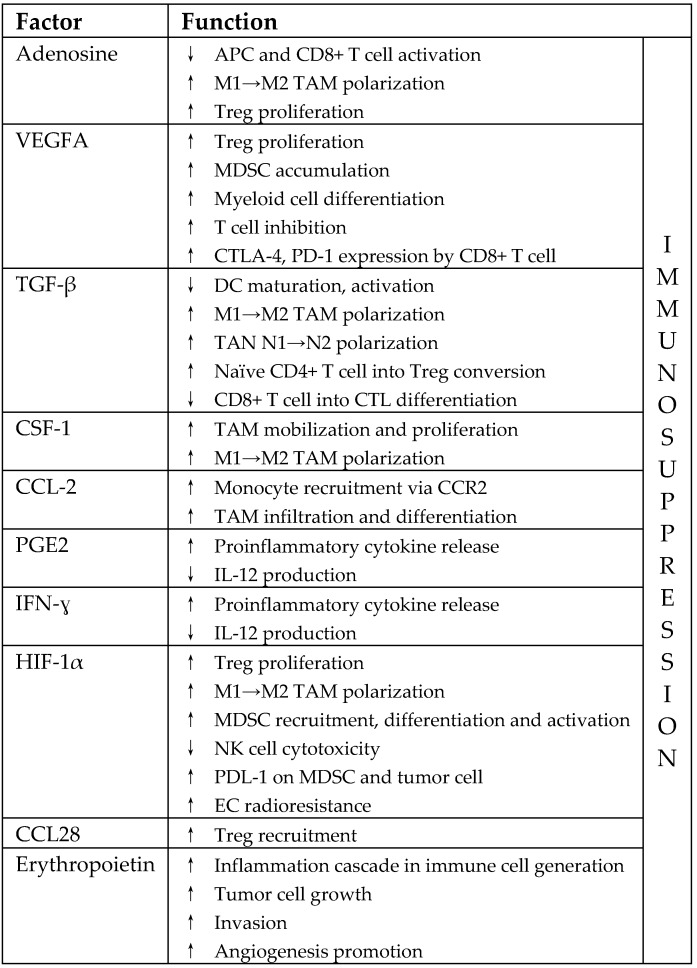
Factors activated in the TME in respose to RT that contribute to tumor radioresistance. VEGFA, vascular endothelial growth factor A; TGF-β, transforming growth factor-β; CSF-1, macrophage-colony stimulating factor; CCL2, C-C motif chemokine ligand 2; PGE2, prostaglandin E2; IFN-γ, interferon-γ; HIF-1α, hypoxia-inducible factor 1α; CCL28, C-C motif chemokine ligand 28; APC, antigen presenting cells; CTLA-4, cytotoxic T-lymphocyte-associated protein 4; PD-1, programmed death 1; TAN, tumor-associated neutrophil; CTL, cytotoxic T lymphocyte; CCR2, C-C chemokine receptor type 2; PDL-1, programmed death ligand 1.
